# Accuracy of Determine TB-LAM Ag to detect TB in HIV infected patients associated with diagnostic methods used in Brazilian public health units

**DOI:** 10.1371/journal.pone.0221038

**Published:** 2019-09-24

**Authors:** Aline Benjamin, Solange Cesar Cavalcante, Leda Fátima Jamal, Denise Arakaki-Sanchez, Josué Nazareno de Lima, Jose Henrique Pilotto, Francisco Ivanildo de Oliveira Junior, Tâmara Newman Lobato Souza, Maria Cristina Lourenço, Maeve Brito de Mello, Pedro Emmanuel Alvarenga Americano do Brasil, Draurio Barreira, Valeria Rolla

**Affiliations:** 1 Clinical Reasearch Laboratory on Mycobacteria, National Institute of Infectious Diseases Evandro Chagas, Oswaldo Cruz Foundation, Rio de Janeiro, Brazil; 2 STD/AIDS Reference and Training Center, Health Secretariat of State of São Paulo, Brazil; 3 National Tuberculosis Program, Ministry of Health, Brasilia, Brazil; 4 STD/AIDS Service, Nova Iguaçu General Hospital, Rio de Janeiro, Brazil; 5 Oswaldo Cruz Institute, Oswaldo Cruz Foundation, Rio de Janeiro, Brazil; 6 Institute of Infectious Disease Emilio Ribas, São Paulo, Brazil; 7 Bacteriology and Bioassay Laboratory, National Institute of Infectious Diseases Evandro Chagas, Oswaldo Cruz Foundation, Rio de Janeiro, Brazil; 8 Pan American Health Organization, Washington DC, United States of America; 9 Clinical Research Laboratory on Immunization and Surveillance in Health, National Institute of Infectious Diseases Evandro Chagas, Oswaldo Cruz Foundation, Rio de Janeiro, Brazil; 10 Unitaid, Geneva, Switzerland; Institute of Tropical Medicine Antwerp, BELGIUM

## Abstract

**Background:**

Determine TB-LAM Ag (LAM) is a point of care test developed to diagnose tuberculosis (TB). The aim of this study was to evaluate the diagnostic performance of LAM in people living with HIV using Brazilian public health network algorithm for TB diagnosis.

**Methods and findings:**

A cross-sectional study design was used to enroll 199 adult patients in two sites in Rio de Janeiro and two in São Paulo. The study enrolled HIV-infected patients with CD4 counts ≤200 cells/mm^3^ (in the Alere PIMA CD4 assay at study screening), patients coughing for at least 2 weeks or presenting a chest radiography suggestive of TB. LAM, in conjunction with sputum smear microscopy or Xpert MTB/RIF (Xpert) as compared to *Mycobacterium tuberculosis* culture, which was used as a reference standard. TB prevalence was 24.6%. Overall accuracy of LAM was 79.9% (73.8%-84.9%), positive and negative predictive values were 62.2% (46.1%-75.9%) and 84% (77.5%-88.8%), respectively. The overall LAM sensitivity was 46.9% (33.7%-60.6%) and specificity was 90.7% (84.9%-94.4%). The best performance of LAM was observed among patients with CD4 counts ≤50 cells/mm^3^ (sensitivity = 70.4% and specificity = 85.9%). When 2 respiratory smears were used in conjunction with LAM, sensitivity increased 22%, as compared to just 2 smears. Furthermore, LAM when used in conjunction with two respiratory smears, was as sensitive as compared to a single one. However, no improvement in TB diagnosis occurred when LAM was used with Xpert as compared to Xpert alone. Among 14 LAM false positive tests, Non-Tuberculosis Mycobacteria were isolated in three cases.

**Conclusion:**

LAM is a point of care test that increased TB diagnosis in immunosuppressed HIV-infected patients when used in conjunction with smear microscopy, but not when used with Xpert in Brazilian public health network sites. Use of LAM test should be considered in settings where immunosuppressed HIV patients need rapid TB diagnosis.

## Introduction

Tuberculosis (TB) diagnosis continues to be a challenge in people living with HIV (PLHIV) since immunosuppression results in atypical images on the chest X-ray [1,2] and paucibacillary disease. This often leads to negative smear microscopy [3,4] and delayed diagnosis as *Mycobacterium tuberculosis* (Mtb) culture is slow to grow.

Brazil used a TB diagnostic algorithm based on smear microscopy and Mycobacterial culture until 2014 [5], when it was modified to incorporate Xpert MTB/RIF (Xpert) into the public health system [6]. Since then, for PLHIV, Brazilian public health laboratories use either: a) Xpert, culture and drugs sensitivity tests (DST) for new TB cases or b) two sputum smears, Xpert, culture and DST for retreatment of TB cases.

Determine TB LAM Ag (LAM) detects lipoarabinomannam, a glycolipid from Mycobacteria wall that is eliminated in the patient’s urine. The overall sensitivity is low [7,8], but the test does increase TB diagnosis in HIV immunosuppressed patients [9]. The World Health Organization (WHO) advises that LAM should only be used on PLHIV with symptoms suggestive of pulmonary and/or extrapulmonary TB, with CD4 ≤100 cells/mm^3^ or in those seriously ill if CD4 cell counts are unavailable [10].

The aim of this study was to evaluate the diagnostic performance of LAM in PLHIV with CD4 counts ≤200 cell/mm^3^, when used in conjunction with smear microscopy or Xpert, compared to Mtb culture to determine if LAM should be incorporated into the Brazilian TB diagnosis algorithm for PLHIV in the public health system.

## Materials and methods

### Ethics statement

This study was approved by the following Institutional Review Boards: National Institute of Infectious Diseases Evandro Chagas, Center for Reference and Training STD/AIDS, Nova Iguaçu General Hospital and Institute of Infectious Disease Emilio Ribas and by National Research Ethics Committee (*CAAE number 439*.*001*).

### Study design

This was a cross-sectional, pragmatic multicenter study with consecutive inclusion of patients suspected of TB attended at 2 sites in Rio de Janeiro (National Institute of Infectious Diseases Evandro Chagas and Nova Iguaçu General Hospital) and 2 sites in São Paulo (Institute of Infectious Disease Emilio Ribas and Center for Reference and Training STD/AIDS). All four sites are public HIV reference health units.

### Participants

Patients were initially screened to detect signs and symptoms of TB and HIV. Chest X-ray was requested for those without a previous exam in the last 15 days before enrollment. Participants were also submitted to a rapid CD4 cell count through Alere PIMA Analyzer. At least one respiratory sample was provided to perform smear microscopy and Mycobacterial culture. Hemoculture and other specimens cultures were done according to patients’ signs and symptoms. During this study, Xpert was incorporated into Brazilian TB diagnosis algorithm and was included in the protocol after an amendment to the original one.

Patients were enrolled if they were 18 years old or more, HIV-positive, signed an informed consent, had cough for at least 2 weeks or presented a chest radiography suspected of TB and had CD4 counts ≤200 cells/mm^3^. Patients were excluded if they were pregnant, did not provide urine, failed to provide at least one respiratory sample or had initiated TB treatment 30 days before study entry.

### Study procedures

All enrolled participants provided a fresh urine sample for LAM detection. The results were independently read by two nurses and photos were taken for future comparisons.

Chest X-rays showing upper lobe opacity, lower lobe upper segment opacity, milliary pattern, mediastinal adenopathy, pleural effusion or cavitation were considered suggestive of TB.

TB diagnostic tests were performed by a certified public bacteriology laboratory for each site involved. Respiratory samples, as well as other suspected site samples, were processed following their own standardized practices. Laboratories staffs were blinded to patient’s clinical data and LAM results. Acid fast stain were done using Ziehl-Neelsen method and Mycobacteria load was reported as negative, scarce bacilli, +, ++ and +++ according to Brazilian TB Guidelines [11]. At the Rio de Janeiro sites, Mycobacterial culture was performed using Lowenstein-Jensen medium and MGIT Mycobacterial Growth Indicator Tubes (BD Diagnostics, USA), while at São Paulo sites only MGIT was performed. Xpert, LAM and Alere PIMA CD4 assays were performed according to manufacturers’ instructional package inserts. Respiratory sample diagnostic routine and LAM test were performed in parallel.

A patient was considered with TB diagnosis if any culture (LJ or MGIT) of any respiratory or no respiratory sample resulted in Mtb isolation.

All clinical and laboratorial data were recorded through Research Electronic Data Capture and data was analyzed using R-project, version 3.4.3. Descriptive analyzes were used for socio-demographic and clinical characteristics. Diagnostic accuracy measurements of sensitivity, specificity and predictive values were estimated with 95% confidence interval in overall population and stratified by CD4 cell counts (≤50, 51–100 and 101–200), with Mtb culture as the reference standard. The tests combinations were conducted in parallel, which means subjects were classified as positive when at least one of them was positive, even if one of the tests was absent.

### Sample size calculation

Since there was little information regarding TB diagnosis when adding LAM to existing testing algorithms, the sample size for the study was based on several considerations. Since smear sensitivity was expected to be between 50% and 60% an increase of sensitivity between 20% and 40% was expected when adding LAM to smear. At a parsimonia situation, a non-overlapping 95% binomial confidence interval could be observed when a sensitivity increases from 55% to 85% and tested with a sample size of 40. As the inclusion strategy was to be consecutive, and TB prevalence was expected to be between 30% and 40% at the lower CD4 strata, a reasonable sample size would be between 100 and 133.

Although Xpert was unavailable at Brazil in the beginning of the study, its’ design allowed the analyses of this test in TB diagnosis algorithm.

## Results

There were 278 patients screened and 199 enrolled between August, 2014 and December, 2015. Among those not eligible 25 were HIV negative, one had no cough neither suspected chest X-ray, 45 had CD4 counts >200 cells/mm^3^, one could not provide urine, six did not provide a respiratory sample and one used TB drugs previously (S1 Fig). Of the 199 enrolled patients, 127 were men and 72 were women, the mean (standard deviation) age was 39.06 (±9.95) years and the median CD4 count was 42 (IQR 19–82) cells/mm^3^ at enrollment. There was no difference regarding clinical characteristics between LAM negative and positive patients, except for CD4 counts. Most LAM positive patients (83.78%) had a CD4 count ≤50 cells/mm^3^ (Table 1). Eighty-nine patients presented chest images suggestive of TB and only 8/89 presented cavitation–the most typical pulmonary abnormality observed in TB. Among these 8 patients, 2 had CD4 ≤50 cells/mm^3^, 2 had CD4 between 51 and 100 cells/mm^3^ and the other 4 had CD4 between 101 and 200 cells/mm^3^.

**Table 1:** Absolute and relative frequencies of clinical characteristics at TB investigation by LAM result (relative frequencies are in parenthesis, except as otherwise indicated).

	LAM Negative	LAM Positive	Total	Statistic Test	P Value
**Total**	162	37	199		
**Gender**					
Male	100 (61.73)	27 (72.97)	127 (63.82)	Chisq. (1 df) = 1.198	0.274
Female	62 (38.27)	10 (27.03)	72 (36.18)		
**Age**					
Mean (SD)	39.76 (10.01)	36.00 (9.16)	39.06 (9.95)	t-test (198 df) = 2.090	0.038
**CD4** counts median (IQR)	51 (22.3,90.8)	25 (15,39)	42 (19,82)	Rannksum test	< 0.001
**CD4**					
[0,50]	81 (50.00)	31 (83.78)	112 (56.28)	Chi-square (2df) = 14.603	< 0.001
(50,100]	47 (29.01)	5 (13.51)	52 (26.13)		
(100,200]	34 (20.99)	1 (2.70)	35 (17.59)		
**Chest Xray** **suggestive of TB?**					
No	66 (43.42)	12 (34.29)	78 (41.71)	Chi-square (2df) = 2.961	0.228
Yes	68 (44.74)	21 (60.00)	89 (47.59)		
Inespecific abnormalities	18 (11.84)	2 (5.71)	20 (10.70)		
**Night sweats since beginning** **of symptoms?**					
No	66 (40.74)	10 (27.03)	76 (38.19)	Fisher's exact test	0.137
Yes	96 (59.26)	27 (72.97)	123 (61.81)		
**Dyspnoea since beginning** **of symptoms?**					
No	46 (28.40)	10 (27.03)	56 (28.14)	Fisher's exact test	1
Yes	116 (71.60)	27 (72.97)	143 (71.86)		
**Cough since beginning** **of symptoms?**					
No	9 (5.56)	4 (10.81)	13 (6.53)	Fisher's exact	0.268
Yes	153 (94.44)	33 (89.19)	186 (93.47)		
**Hemoptysis since beginning** **of symptoms?**					
No	134 (82.72)	33 (89.19)	167 (83.92)	Fisher's exact	0.745
Yes	24 (14.81)	4 (10.81)	28 (14.07)		
Don´t know	4 (2.47)	0 (0.00)	4 (2.01)		
**Chest pain since beginning** **of symptoms?**					
No	68 (41.98)	17 (45.95)	85 (42.71)	Fisher's exact	0.768
Yes	93 (57.41)	20 (54.05)	113 (56.78)		
Don´t know	1 (0.62)	0 (0.00)	1 (0.50)		
**History of TB or** **TB previous treatment?**					
No	121 (74.69)	30 (81.08)	151 (75.88)	Fisher's exact	0.779
Yes	39 (24.07)	7 (18.92)	46 (23.12)		
Don´t know	2 (1.23)	0 (0.00)	2 (1.01)		
**TB clinical form**					
Pulmonary	18 (11.11)	16 (43.24)	34 (17.09)	Fisher's exact	0.590
Pleuropulmonary	2 (1.23)	0 (0.00)	2 (1.01)		
Disseminated	6 (3.70)	7 (18.92)	13 (6.53)		
Extrapulmonary	0 (0.00)	0 (0.00)	0 (0.00)		

Most patient could provide spontaneous sputum for the first respiratory sample, but 54.3% didn´t collected the second sample. More detailed information about diagnostic tests are in S1 Table.

**Fig 1:**
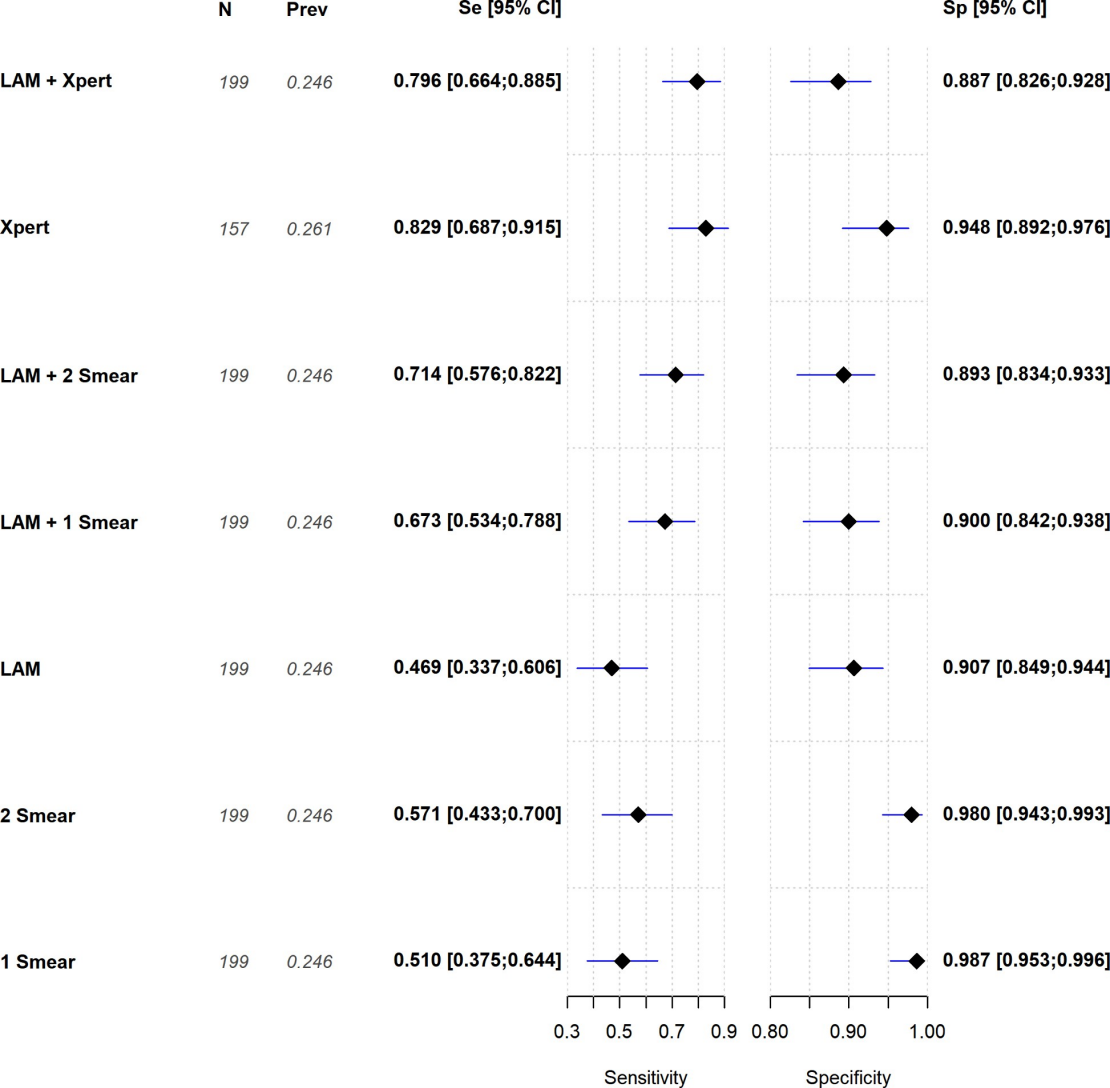
Forest plot with sensitivity and specificity for different combination of tests in overall population.

TB prevalence was 18.6% and 24.6% according to LAM and Mtb culture, respectively. LAM’s overall accuracy was 79.9% (73.8%-84.9%). Positive and negative predictive values were 62.2% (46.1%-75.9%) and 84% (77.5%-88.8%), respectively. The overall sensitivity and specificity of LAM was 46.9% (33.7%-60.6%) and 90.7% (84.9%-94.4%), respectively (Fig 1).

**Fig 2:**
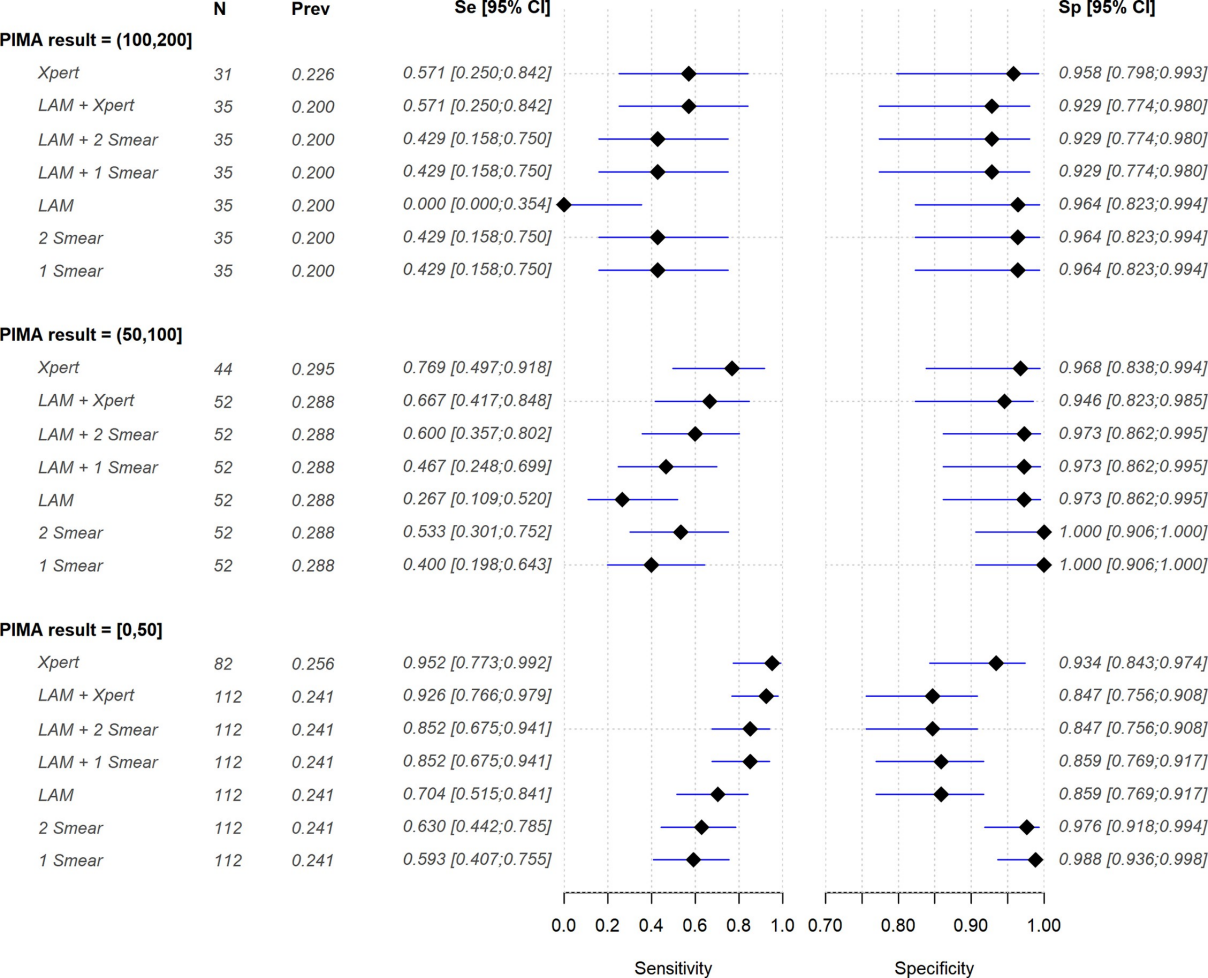
Forest plot with sensitivity and specificity for different combination of tests in different strata of CD4.

The best performance of LAM was observed among patients with CD4 counts ≤50 cells/mm^3^, where sensitivity was 70.4% (51.5%-84.1%) and specificity was 85.9% (76.9%-91.7%). When two smears microscopy were combined with LAM, sensitivity was increased by 22% when compared to two smears alone, achieving a sensitivity close to Xpert. Furthermore, LAM combined with two smears was as sensitive as when combined to a single smear. LAM did not add sensitivity when combined to Xpert when compared to Xpert alone (Fig 2). For patients with higher CD4 cell counts, LAM’s sensitivity was too low to explore combinations either with Xpert or sputum smear.

There were 14 false positive LAM results. In three cases, Non-Tuberculosis Mycobacteria grew in culture.

## Discussion

In our study, the accuracy of LAM was low in overall population, but was better in patients with low CD4 cell counts, as already reported [9,12–14]. The greatest benefit of LAM was observed in patients with CD4 counts ≤50 cells/mm^3^. LAM increased TB diagnosis when associated with smear microscopy, achieving a sensitivity close to Xpert, indicating what could be an interesting option for settings with poor diagnosis infrastructure. In addition, LAM could be combined with a single smear, without decreasing sensitivity, speeding up the diagnosis. LAM did not improve TB diagnosis when associated with Xpert, as observed by other authors [9]. However, Xpert was included in the Brazilian diagnosis algorithm during the study and only 73.2% of those with CD4 ≤50 cells/mm^3^ were tested for Xpert. The lower number of cases could have influenced these analyses.

One of the pillars for decreasing TB incidence is the rapid diagnosis [15]. Even today, a timely diagnosis is a limiting factor for early detection of TB, especially in developing countries where it is difficult for established laboratories to perform Mtb culture for high burden locations. Therefore, in many countries, the TB diagnosis is still based on smears and chest X-rays, which have low sensitivity and specificity. In PLHIV, the diagnosis is even more complex, since immunosuppression prevents the formation of granuloma, reducing the pulmonary cavities, resulting in atypical chest images [1,2]. Additionally, immunosuppressed individuals are paucibacillary and therefore more susceptible to have negative smear microscopy [3,4], which may lead to empirical or misleading treatments. Indeed, we observed a low number of patients with pulmonary cavity and a high number of negative smears in our study. Thus, the rapid TB diagnosis in immunodeficient patients is still a challenge.

Xpert was a breakthrough in TB diagnosis due to its rapid and high accuracy, thereby decreasing time to initiate treatment [16]. Although Brazilian TB diagnosis algorithms have included Xpert since 2014, this test is not available throughout the country and is not a real point of care (PoC) test as most equipment are located in Mycobacteria laboratories. The most important criteria to allocate GeneXpert was regional TB incidence. Therefore, the machine was first provided to 92 municipalities. Additionally, nine others were provided for research purposes [17]. In low TB burden areas, smear microscopy is still the first method to diagnose TB in PLHIV and definitive diagnosis remains based on culture results.

There are limitations to making Xpert easily available in many health units. Xpert requires stable electric power and air conditioning, which can be an obstacle in low income countries. After one year of implementation, electrical power problems and local high temperature were common problems in Brazil [17]. In order to improve TB diagnosis in poor settings, a real” PoC” test is one which is quick, easy to perform and that could be used close to patients. LAM combined with smear microscopy could increase TB diagnosis in Brazil, as Xpert is not fully available in all health facilities. In addition, urine is an easy to get specimen and LAM does not need biosafety protocols. It can even be performed at the bedside allowing for a TB diagnosis in different settings where PLHIV access health care. As PLHIV usually have difficulties in providing sputum, urine becomes a suitable biological sample for TB testing [3].

Severely immunodeficient patients may also have disease caused by NTM more frequently and there is a similarity between Mtb and other Mycobacteria lipoarabinomannam. Therefore, attention must be paid to cases of NTM diagnosed with a positive LAM. In this study, NTM were detected in three patients with positive LAM results. As we know, Mycobacterial cultures have limitations and can be affected by inadequate or insufficient sample, especially when patient has already started TB treatment. Therefore, positive LAM tests without a positive culture should be considered in some circumstances. In addition, recent studies had shown that LAM could potentially reduce mortality in PLHIV admitted to hospitals [18,19] due to an increase in TB diagnosis.

Our study has limitations as Xpert was included in the protocol latter, reducing the chance to fully observe its behavior in Brazilian algorithm. However, our straightness was to include more than 50% participants with very low CD4 counts allowing a good analysis on LAM performance in this specific group.

In conclusion, LAM is a real PoC test and should be used in combination with smear microscopy for TB diagnosis in immunosuppressed PLHIV. If CD4 counts are unknown, some clinical characteristics are indicative of immunosuppression and could predict a low CD4 count, being helpful to select which patients should be tested.

Although there have been previous Brazilian studies about LAM in leprosy [20,21] this is the first study using Determine TB LAM Ag conducted in Brazil using the national algorithm in established clinical sites demonstrating that LAM should be used in sites with high burden of HIV to improve the rapid diagnosis of TB.

## Supporting information

S1 Fig STARD flow diagram. Patient flow and Mtb culture (reference test) result.
(DOCX)

S1 Table Absolute and relative frequencies of diagnostic tests at TB investigation by LAM result. (DOCX)

S1 File STARD checklist. (DOCX)

S2 File Data_LAM_final. (CSV)

S3 File Data_LAM_final. (ZIP)

S4 File Describe_LAM_final. (TXT)
