# Supplementary material for: Accuracy of Determine TB-LAM Ag to detect TB in HIV infected patients associated with diagnostic methods used in Brazilian public health units
Source: PLoS One. 2019 Sep 24;14(9):e0221038. doi: 10.1371/journal.pone.0221038 (PMC6759169; doi:10.1371/journal.pone.0221038)
Supplement: S1 Fig — Patient flow and Mtb culture (reference test) result. (DOCX) [file pone.0221038.s001.docx]

**S1 Fig. STARD flow diagram.** Patient flow and Mtb culture (reference test) result.

Mtb Culture Negative

n = 136

Mtb Culture Positive

n = 26

Mtb Culture Negative

n = 14

Mtb Culture Positive

n = 23

LAM Negative

n = 162

LAM Positive

n = 37

Potentially elegible participants

n = 278

Elegible participants

n = 199

Excluded

HIV negative (n = 25)

No cough or suspected Xray (n = 1)

CD4 > 200 (n = 45)

No urine sample (n = 1)

No respiratory sample (n = 6)

Previous TB treatment (n = 1)

LAM Test (Index)

n = 199
