# Supplementary material for: Accuracy of Determine TB-LAM Ag to detect TB in HIV infected patients associated with diagnostic methods used in Brazilian public health units
Source: PLoS One. 2019 Sep 24;14(9):e0221038. doi: 10.1371/journal.pone.0221038 (PMC6759169; doi:10.1371/journal.pone.0221038)
Supplement: S1 Table — (DOCX) [file pone.0221038.s002.docx]

**S1** **Table Absolute and relative frequencies of diagnostic tests at TB investigation by LAM result (relative frequencies are in parenthesis).** Xpert was introduced during the study and were performed in 157 participants.

|  | **LAM  Negative** | **LAM  Positive** | **Total** | **Statistic Test** | **P Value** |
| --- | --- | --- | --- | --- | --- |
| **Total** | 162 | 37 | 199 |  |  |
| **Sputum collection**  **(sample 1)** |  |  |  |  |  |
| Spontaneous | 116 (71.60) | 21 (56.76) | 137 (68.84) | Fisher's exact | 0.056 |
| Induced | 42 (25.93) | 14 (37.84) | 56 (28.14) |  |  |
| BAL | 1 (0.62) | 2 (5.41) | 3 (1.51) |  |  |
| Tracheal secretion | 3 (1.85) | 0 (0.00) | 3 (1.51) |  |  |
| **Sputum collection**  **(sample 2)** |  |  |  |  |  |
| Spontaneous | 65 (40.12) | 14 (37.84) | 79 (39.70) | Fisher's exact | 0.853 |
| Induced | 5 (3.09) | 0 (0.00) | 5 (2.51) |  |  |
| BAL | 3 (1.85) | 0 (0.00) | 3 (1.51) |  |  |
| Tracheal secretion | 3 (1.85) | 1 (2.70) | 4 (2.01) |  |  |
| Not done | 86 (53.09) | 22 (59.46) | 108 (54.27) |  |  |
| **Sputum smear result**  **(sample 1)** |  |  |  |  |  |
| Negative | 151 (93.21) | 21 (56.76) | 172 (86.43) | Fisher's exact | < 0.001 |
| Rare bacillus | 1 (0.62) | 4 (10.81) | 5 (2.51) |  |  |
| Positive + | 3 (1.85) | 6 (16.22) | 9 (4.52) |  |  |
| Positive ++ | 2 (1.23) | 5 (13.51) | 7 (3.52) |  |  |
| Positive +++ | 5 (3.09) | 1 (2.70) | 6 (3.02) |  |  |
| **Sputum smear result**  **(sample 2)** |  |  |  |  |  |
| Negative | 69 (90.79) | 10 (66.67) | 79 (86.81) | Fisher's exact | 0.018 |
| Rare bacillus | 1 (1.32) | 0 (0.00) | 1 (1.10) |  |  |
| Positive + | 3 (3.95) | 1 (6.67) | 4 (4.40) |  |  |
| Positive ++ | 2 (2.63) | 1 (6.67) | 3 (3.30) |  |  |
| Positive +++ | 1 (1.32) | 3 (20.00) | 4 (4.40) |  |  |
| **Xpert® MTB/RIF** |  |  |  |  |  |
| Negative | 110 (73.33) | 7 (20.59) | 117 (63.59) | Fisher's exact test | < 0.001 |
| Positive | 19 (12.67) | 21 (61.76) | 40 (21.74) |  |  |
| Not done | 0 (0.00) | 0 (0.00) | 0 (0.00) |  |  |
| **Sputum culture result**  **(sample 1)** |  |  |  |  |  |
| Negative | 107 (66.88) | 10 (27.03) | 117 (59.39) | Fisher's exact test | < 0.001 |
| MTB | 20 (12.50) | 22 (59.46) | 42 (21.32) |  |  |
| Other mycobacteria | 5 (3.12) | 3 (8.11) | 8 (4.06) |  |  |
| Contaminated | 28 (17.50) | 2 (5.41) | 30 (15.23) |  |  |
| **Sputum culture result (sample 2)** |  |  |  |  |  |
| Negative | 49 (30.25) | 4 (10.81) | 53 (26.63) | Fisher's exact test | 0.020 |
| MTB | 11 (6.79) | 5 (13.51) | 16 (8.04) |  |  |
| Other mycobacteria | 0 (0.00) | 1 (2.70) | 1 (0.50) |  |  |
| Contaminated | 11 (6.79) | 2 (5.41) | 13 (6.53) |  |  |
| Not done | 91 (56.17) | 25 (67.57) | 116 (58.29) |  |  |
| **Blood culture result**  **(sample 1)** |  |  |  |  |  |
| Negative | 147 (91.88) | 26 (70.27) | 173 (87.82) | Fisher's exact test | < 0.001 |
| MTB | 0 (0.00) | 8 (21.62) | 8 (4.06) |  |  |
| Other mycobacteria | 0 (0.00) | 2 (5.41) | 2 (1.02) |  |  |
| Contaminated | 2 (1.25) | 0 (0.00) | 2 (1.02) |  |  |
| Not done | 11 (6.88) | 1 (2.70) | 12 (6.09) |  |  |
|  |  |  |  |  |  |
| **Blood culture result**  **(sample 2)** |  |  |  |  |  |
| Negative | 94 (58.75) | 12 (33.33) | 106 (54.08) | Fisher's exact test | < 0.001 |
| MTB | 0 (0.00) | 5 (13.89) | 5 (2.55) |  |  |
| Other mycobacteria | 0 (0.00) | 0 (0.00) | 0 (0.00) |  |  |
| Contaminated | 1 (0.62) | 0 (0.00) | 1 (0.51) |  |  |
| Not done | 65 (40.62) | 19 (52.78) | 84 (42.86) |  |  |
|  |  |  |  |  |  |
| **Blood culture result**  **(sample 3)** |  |  |  |  |  |
| Negative | 85 (53.12) | 11 (29.73) | 96 (48.73) | Fisher's exact test | < 0.001 |
| MTB | 1 (0.62) | 4 (10.81) | 5 (2.54) |  |  |
| Other mycobacteria | 0 (0.00) | 0 (0.00) | 0 (0.00) |  |  |
| Contaminated | 0 (0.00) | 0 (0.00) | 0 (0.00) |  |  |
| Not done | 74 (46.25) | 22 (59.46) | 96 (48.73) |  |  |
| **Specimen from other sites** |  |  |  |  |  |
| Ganglionic | 0 (0.00) | 0 (0.00) | 0 (0.00) | Fisher's exact test | 0.043 |
| Pleural | 1 (0.62) | 0 (0.00) | 1 (0.50) |  |  |
| Liquids | 2 (1.23) | 3 (8.11) | 5 (2.51) |  |  |
| Other biopsies | 1 (0.62) | 1 (2.70) | 2 (1.01) |  |  |
| Not done | 158 (97.53) | 33 (89.19) | 191 (95.98) |  |  |
| **Results from other sites** |  |  |  |  |  |
| Negative | 3 (75.00) | 3 (75.00) | 6 (75.00) | Fisher's exact test | 1.000 |
| MTB | 1 (25.00) | 0 (0.00) | 1 (12.50) |  |  |
| Other mycobacteria | 0 (0.00) | 1 (25.00) | 1 (12.50) |  |  |
| Contaminated | 0 (0.00) | 0 (0.00) | 0 (0.00) |  |  |
